# Total Hip Arthroplasty for Post‐Traumatic Hip Arthritis in Chronic Pubic Diastasis: A Case Report

**DOI:** 10.1155/cro/8930297

**Published:** 2026-06-27

**Authors:** Avtar Singh Kamboj, Abhijit Das, Babaji Sitaram Thorat, Arcot Reddy Vamsi Krishna, Kshitij Srivastav, Arshid H. Wani, Prabhat Mittal

**Affiliations:** ^1^ Department of Orthopaedics, Amandeep Hospitals, Amritsar, Punjab, India

**Keywords:** acetabular orientation, acetabular retroversion, Harris Hip Score, pelvic malrotation, post-traumatic arthritis, pubic diastasis, total hip arthroplasty

## Abstract

**Introduction:**

Total hip arthroplasty (THA) in the presence of chronic pubic diastasis is uncommon and technically demanding due to hemipelvic malrotation and abnormal acetabular orientation.

**Case Presentation:**

A 41‐year‐old woman developed advanced post‐traumatic hip osteoarthritis following fixation of an acetabular fracture and sacroiliac joint injury, complicated by a 90‐mm chronic pubic diastasis. Preoperative imaging showed a vertically oriented, retroverted acetabulum with external hemipelvic rotation. THA was performed using a modified posterolateral approach with normalized imaging and landmark‐based acetabular orientation. A press‐fit uncemented acetabular component with a dual‐mobility liner and a short uncemented femoral stem were implanted. The postoperative course was uneventful. The Harris Hip Score improved from 74 at 2 weeks to 93 at 12 weeks, and radiographs up to 2 years confirmed stable osseointegration.

**Conclusion:**

This case highlights technical strategies for performing THA in post‐traumatic pubic diastasis with prior fixation, emphasizing functional pelvic normalization and reproducible landmark‐based acetabular orientation. This report was prepared in accordance with CARE guidelines.

## 1. Introduction

Total hip arthroplasty (THA) is a well‐established and durable procedure for the management of degenerative hip disease, providing reliable pain relief and functional improvement [1]. However, congenital or acquired pelvic deformities substantially increase the technical complexity of THA by altering acetabular orientation, pelvic biomechanics, and the anatomical landmarks required for accurate component placement [2, 3]. Such challenges are well recognized in conditions including developmental dysplasia of the hip and in patients with prior trauma or reconstructive pelvic surgery [4–6].

Post‐traumatic pubic diastasis is rarely encountered during late reconstructive procedures, as most pelvic ring injuries are managed acutely with reduction and stabilization [7]. When present chronically, pubic diastasis is often associated with hemipelvic malrotation, acetabular retroversion, and dysplastic changes that complicate restoration of the native hip center during THA [8, 9]. These deformities may lead to superior or lateral migration of the femoral head, secondary coxarthrosis, and limb length discrepancy due to pelvic asymmetry. The literature addressing THA in this setting remains limited.

This case adds to the limited literature on THA in pubic diastasis by describing a severe post‐traumatic deformity (90‐mm diastasis) with prior acetabular and sacroiliac fixation, and highlights a reproducible strategy using pelvic normalization, landmark‐guided cup positioning, and dual mobility with short‐stem fixation.

## 2. Case Presentation

A 41‐year‐old woman presented with progressive right hip pain and worsening difficulty in ambulation over 6 months. She had previously sustained a right acetabular fracture with an associated sacroiliac joint injury, which had been treated 8 months earlier with posterior wall acetabular plating and sacroiliac screw fixation. At presentation, she was able to walk short distances with a walker and demonstrated a painful, antalgic gait.

Clinical examination revealed marked restriction of right hip motion, with flexion limited to 50°, external rotation to 30°, internal rotation to 10°, and abduction/adduction to 10°. The limb showed fixed external rotation posture of the limb with an estimated true shortening of approximately 3 cm. Erythrocyte sedimentation rate (ESR), C‐reactive protein (CRP), and total leukocyte count were normal, with no radiographic signs of infection such as implant loosening, periosteal reaction, or osteolysis. Given the low clinical suspicion and normal investigations, joint aspiration and further invasive evaluation were not considered necessary. Rapid progression was likely multifactorial due to post‐traumatic cartilage damage, altered load transmission from hemipelvic malrotation, femoral head subluxation, and worsening functional limitation over 6 months.

**Figure 1:**
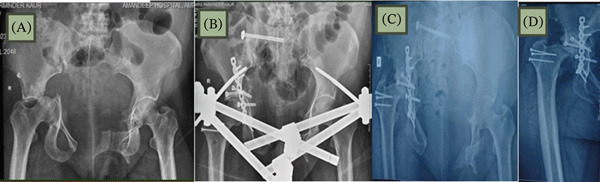
(A, B) Preoperative radiographs demonstrating right‐sided acetabular fracture with hip dislocation and sacroiliac injury treated with posterior wall plating, sacroiliac screw fixation, and a supra‐acetabular external fixator. (C, D) Normalized preoperative AP view with 20° internal rotation showing advanced hip osteoarthritis with femoral head deformity and subluxation, chronic pubic diastasis, and a Dorr type A femur.

**Figure 2:**
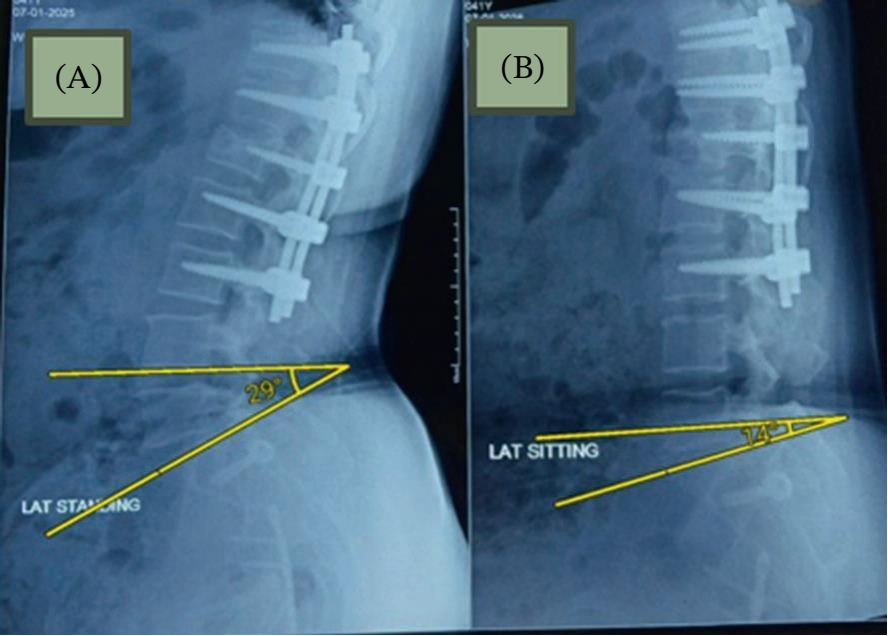
Lateral radiographs of the lumbosacral spine obtained in the (A) standing and (B) sitting positions demonstrating preserved spinopelvic mobility.

Around 20° of internal rotation was applied intraoperatively, with fluoroscopic confirmation of symmetric obturator foramina and proper alignment of the sacrococcygeal junction over the pubic symphysis. This maneuver restored functional pelvic orientation and corrected the hemipelvic malrotation. Radiographs showed advanced post‐traumatic osteoarthritis with femoral head deformity, subluxation, retained implants, and 90‐mm pubic diastasis (Figure 1). Full‐length femoral radiographs showed a Dorr type A femur. Dynamic lateral radiographs of the lumbosacral spine demonstrated a 15° change in sacral slope, consistent with preserved spinopelvic mobility (Figure 2).

**Figure 3:**
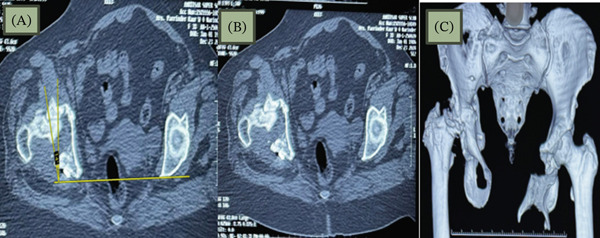
(A–C) CT demonstrating acetabular retroversion of 14°, vertical acetabular orientation, and external rotation of the right hemipelvis.

Computed tomography of the pelvis revealed external rotation of the hemipelvis, a vertically oriented acetabulum, and 14° of acetabular retroversion with associated dysplastic changes (Figure 3). Following comprehensive evaluation and preoperative planning, THA was performed in October 2023 by a senior arthroplasty surgeon (Table 1).

**Table 1:** Patient timeline.

Time	Event
February 2023	Outside done ORIF for acetabular injury and sacroiliac screw
6 months later	Progressive hip pain
October 2023	THA performed
Postop Day 1	Mobilization started
6 weeks follow‐up	Full weight bearing
3 months follow‐up	Independent ambulation
2 years follow‐up	Stable osseointegration

### 2.1. Therapeutic Intervention

The procedure was performed under spinal anesthesia with perioperative administration of tranexamic acid and cefuroxime. The patient was positioned in the lateral decubitus position with slight anterior tilt. A posterolateral approach through a modified Gibson incision was used, with careful identification and protection of the sciatic nerve. The short external rotators were detached, followed by longitudinal capsulotomy and hip dislocation.

#### 2.1.1. Pelvic Normalization Technique

Preoperative CT imaging demonstrated approximately 20° functional external rotation of the affected hemipelvis relative to the contralateral side. Intraoperatively, pelvic normalization was performed before acetabular preparation to establish a functional coronal reference plane. The affected lower limb was internally rotated gradually under fluoroscopic guidance until the following criteria were met:

1. Bilateral obturator foramina appeared symmetric on AP fluoroscopy.
2. The sacrococcygeal junction aligned vertically over the pubic symphysis.
3. Iliac wing asymmetry was minimized.
4. The transverse acetabular ligament and acetabular walls corresponded with the corrected fluoroscopic orientation.

Approximately 20° of internal rotation was required to achieve these criteria, consistent with preoperative CT estimation of hemipelvic malrotation. After normalization, acetabular reaming and cup positioning were performed relative to this corrected functional pelvic plane rather than the distorted native hemipelvis. Reproducibility was confirmed by repeated fluoroscopic assessment before definitive component implantation.

After femoral head removal, the native acetabulum was identified using preserved bony landmarks, including the anterior and posterior walls and the transverse acetabular ligament, with additional guidance from preoperative imaging. Sequential reaming was performed to restore the anatomic hip center based on a normalized AP orientation. Acetabular inclination and anteversion were set using mechanical guides and confirmed on postoperative radiographs; reported anteversion refers to radiographic anteversion. A press‐fit uncemented acetabular cup (52 mm; made of titanium alloy with porous coating and hydroxyapatite coating) was implanted with appropriate inclination and anteversion, followed by insertion of a modular dual‐mobility liner.

Femoral preparation was completed with sequential broaching. Femoral anteversion (~15°) was set using posterior condylar alignment and intraoperative cues, with trial reduction confirming leg length, offset, and stability. An uncemented short lateralized femoral stem (size 2) was implanted, followed by insertion of a 28‐mm ceramic femoral head. Trial reduction confirmed restoration of the hip center and femoral offset. Radiographic assessment demonstrated restoration of the hip center to within a few millimeters of the contralateral side. The preoperative limb‐length discrepancy of approximately 3 cm was corrected to less than 5 mm postoperatively. Combined anteversion was estimated intraoperatively to be within functional safe ranges. Dual‐mobility articulation was specifically selected to reduce instability risk in the setting of functional acetabular malversion due to hemipelvic rotation. The short stem allowed intraoperative adjustment of femoral version and preservation of proximal bone stock in the presence of altered pelvic mechanics. Posterior soft tissues were repaired using the Ranawat technique to enhance stability. The wound was closed in layers over a suction drain [10, 11]. Total operative time was 80 min, with an estimated blood loss (using suction volume minus irrigation and sponge weight) of 200 mL.

### 2.2. Follow‐Up and Outcomes

**Figure 4:**
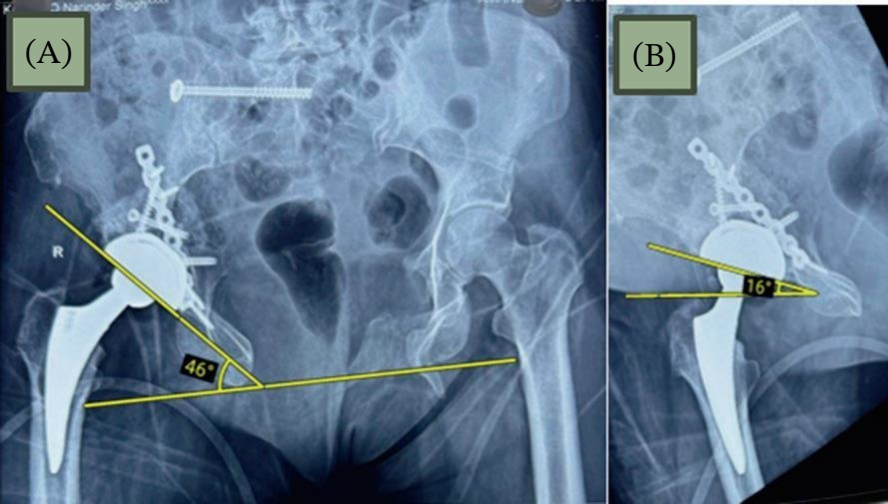
(A, B) Immediate postoperative anteroposterior pelvic radiographs demonstrating satisfactory acetabular component positioning with an inclination of 46° and anteversion of 16°.

Postoperative management included multimodal analgesia and thromboprophylaxis with mechanical calf pumps and low‐molecular‐weight heparin. Routine monitoring revealed no early complications. Immediate postoperative radiographs confirmed satisfactory component orientation, with an acetabular inclination of 46° and anteversion of 16° (Figure 4). Mobilization with partial weight‐bearing and supervised physiotherapy was initiated on the first postoperative day. Intravenous antibiotics were discontinued on Day 1, and the suction drain was removed on Day 2. Postoperatively, enoxaparin 40 mg s.c. was administered for 3 days, followed by aspirin 150 mg for 4 weeks as thromboprophylaxis. The patient was discharged on postoperative Day 5 following an uncomplicated recovery, with suture removal on Day 15.

**Figure 5:**
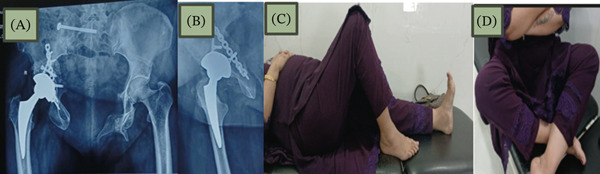
(A, B) Anteroposterior pelvic radiographs at 3‐month follow‐up demonstrating stable osseointegration of the acetabular and femoral components. (C, D) Clinical photographs obtained at 3 months postoperatively showing a good range of motion.

Physiotherapy was progressively advanced, and full weight bearing without assistive devices was achieved by 6 weeks. The patient regained independent ambulation by 8 weeks and returned to routine daily activities by 3 months (Figure 5).

**Figure 6:**
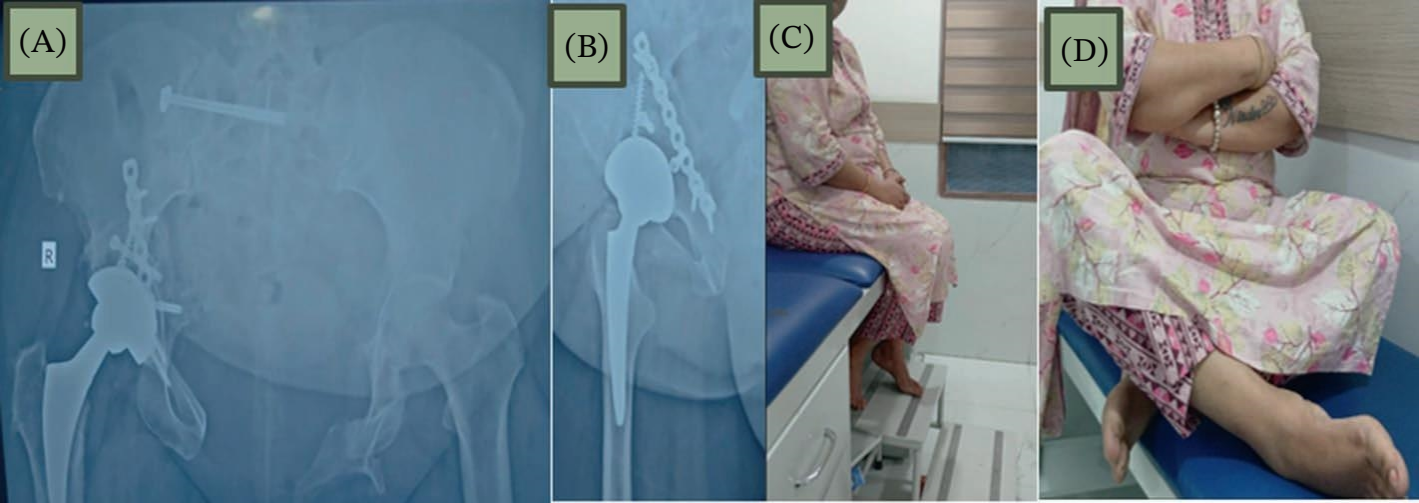
(A, B) Anteroposterior pelvic radiographs at 2‐year follow‐up demonstrating stable osseointegration of the acetabular and femoral components. (C, D) Clinical photographs obtained at 2 years postoperatively showing a good range of motion.

Serial radiographs up to 2 years demonstrated stable osseointegration of both components without loosening or subsidence (Figure 6). The Harris Hip Score improved from 74 at the early postoperative assessment (2 weeks), reflecting baseline postoperative pain relief and early mobility rather than definitive functional recovery, to 93 at 12 weeks, indicating substantial functional improvement [12, 13]. No postoperative complications, including infection, dislocation, deep vein thrombosis/pulmonary embolism or nerve injury, were observed. At 2‐year follow‐up, the patient remained pain‐free without instability or gait aids. Harris Hip Score was maintained at 93. Limb‐length discrepancy remained < 5 mm clinically, and no dislocation, loosening, infection, or revision was observed.

## 3. Discussion

THA in the presence of chronic pubic diastasis remains an uncommon and technically demanding reconstructive scenario. Corrective pelvic procedures such as osteotomy are generally unsuitable in skeletally mature patients because of limited remodeling potential and altered pelvic biomechanics. Pelvic reconstruction was avoided due to the chronic, compensated deformity with preserved spinopelvic mobility. Management focused on restoring hip biomechanics, and isolated THA was considered sufficient, as additional pelvic reconstructive procedures were unlikely to provide meaningful functional benefit [9, 14]. The 2‐week Harris Hip Score mainly reflected early pain relief and initial recovery, whereas the 12‐week and 2‐year scores better represented functional recovery after rehabilitation. Although formal preoperative PROMs were unavailable due to referral after prior trauma surgery, the patient had substantial baseline disability with painful restricted hip motion, walker‐assisted ambulation, limb shortening, antalgic gait, and progressive limitation of daily activities, precluding direct comparison with postoperative functional scores. Despite the limited literature, available evidence suggests that favorable outcomes can be achieved when THA is performed with meticulous preoperative planning and accurate restoration of the hip center within functional safe zones [15, 16]. Restoration of hip biomechanics including hip center, offset, and limb length is critical in such deformities. Chronic pubic diastasis from the initial pelvic injury led to progressive acetabular malorientation and hip degeneration, ultimately necessitating THA. This case emphasizes key technical strategies for stable reconstruction in such complex settings. Unlike previously reported congenital cases, this represents a severe post‐traumatic diastasis (90 mm) with prior acetabular and sacroiliac fixation. Management focused on pelvic normalization, landmark‐guided acetabular orientation, and the combined use of dual‐mobility articulation with short‐stem fixation.

The principal technical challenges arise from distorted pelvic anatomy, including hemipelvic malrotation, acetabular retroversion, vertical acetabular orientation, and associated dysplastic changes, all of which complicate component positioning and stable fixation [17]. In this setting, reliable intraoperative identification of preserved osseous landmarks—such as the transverse acetabular ligament, acetabular walls (anterior and posterior)—is essential for accurate acetabular preparation and restoration of native hip biomechanics [18, 19].

Unlike previous reports (Camera et al. and Goetze et al.), which describe congenital diastasis, our case involved post‐traumatic deformity with prior fixation and significant (90 mm) diastasis. Additionally, the use of normalized pelvic imaging and short‐stem fixation distinguishes this case from prior reports where conventional alignment strategies were used [14, 15, 17]. Similar satisfactory outcomes have been reported following THA in patients with congenital or chronic pubic diastasis when meticulous planning principles are applied [20]. Robotic THA improves the accuracy of acetabular cup positioning and hip center restoration, although short‐term functional advantages remain unproven and its use is limited by cost and technical demands [21]. In chronic pubic diastasis, pelvic malrotation necessitates cup planning on a normalized AP view with 20° of hip internal rotation. In this relatively young patient with preserved proximal femoral bone stock (Dorr type A), a metaphyseal‐fixing short stem was selected to preserve bone stock, promote physiological load transfer, and allow intraoperative adjustment of femoral version in the setting of altered pelvic mechanics. A dual‐mobility articulation was chosen to reduce the increased risk of instability and impingement associated with chronic hemipelvic malrotation and functional acetabular retroversion, as supported by previous studies in high‐risk primary THA [22, 23]. Functional orientation was defined relative to a normalized pelvic plane achieved using 20° internal rotation, ensuring symmetric obturator foramina. Cup inclination and anteversion were then positioned within functional safe zones relative to this corrected pelvic plane, rather than the distorted native hemipelvis (Table 2). This case underscores the importance of prioritizing functional over purely anatomical acetabular orientation in complex pelvic deformities—guiding cup placement according to dynamic pelvic alignment, particularly when hemipelvic rotation is present but spinopelvic mobility is preserved. This report has several limitations, including absence of preoperative functional scoring, single‐case design, and lack of long‐term functional outcome measures beyond 2 years. Therefore, the findings should be interpreted with caution.

**Table 2:** Comparison with various studies.

Author	Etiology	Diastasis	Pelvic deformity	THA strategy	Follow‐up	Outcome
Camera et al. [14]	Congenital	Not specified	Exstrophy related	Conventional THA	Short term	Good
Goetze et al. [15]	Congenital dysplasia	Severe	Dysplastic pelvis	THA	Midterm	Good
Bhimani et al. [17]	Chronic pubic diastasis	Severe	Malrotation	THA	Variable	Acceptable
Our case	Post‐traumatic	90 mm	Hemipelvic rotation + retroversion	Pelvic normalization + landmark‐guided orientation	2 years	Excellent

## 4. Conclusion

THA in patients with chronic pubic diastasis following pelvic trauma is technically challenging because of altered acetabular orientation and hemipelvic malrotation. This case demonstrates that careful preoperative planning using normalized imaging, precise restoration of the hip center, and judicious implant selection—including dual‐mobility articulation and a short femoral stem—can result in stable reconstruction and excellent functional outcomes. THA appears to be a feasible treatment option in selected patients with this complex pelvic deformity.

### 4.1. Learning Points

1. Chronic pubic diastasis can result in functional acetabular malorientation and hemipelvic malrotation, significantly increasing the technical complexity of THA.
2. Careful preoperative planning using functional pelvic normalization, fluoroscopic symmetry, and preserved osseous landmarks is essential for accurate restoration of the hip center in distorted pelvic anatomy.
3. The use of dual‐mobility articulation and bone‐preserving femoral stems can enhance stability and facilitate satisfactory functional outcomes in THA performed for complex post‐traumatic pelvic deformities.

## Author Contributions

Avtar Singh Kamboj: primary surgeon, supervision. Abhijit Das: data collection, manuscript drafting. Babaji Sitaram Thorat: surgical assistance, review. Arcot Reddy Vamsi Krishna: literature review, supervision. Kshitij Srivastav: literature review. Arshid H. Wani: follow‐up, data interpretation. Prabhat Mittal: editing and final approval.

## Funding

No funding was received for this manuscript.

## Ethics Statement

Institutional Ethics Committee approval for publication of this case report was obtained from Amandeep Hospitals (IEC/402/2026 dated 04‐02‐2026). Written informed consent was obtained from the patient.

## Conflicts of Interest

The authors declare no conflicts of interest.

## Patient Perspective

The patient reported substantial pain relief and improvement in walking ability following surgery. She regained independence in daily activities and expressed satisfaction with restoration of mobility and limb function.

## General Statement

This case report has been prepared in accordance with the CARE (CAse REport) guidelines. A completed CARE checklist has been submitted as supplementary material. The manuscript sections including patient information, clinical findings, diagnostic assessment, therapeutic intervention, follow‐up, and patient perspective have been structured in accordance with CARE recommendations.
